# A New Outlook on the Ability to Accumulate an Iodine Contrast Agent in Solid Lung Tumors Based on Virtual Monochromatic Images in Dual Energy Computed Tomography (DECT): Analysis in Two Phases of Contrast Enhancement

**DOI:** 10.3390/jcm10091870

**Published:** 2021-04-26

**Authors:** Arkadiusz Zegadło, Magdalena Żabicka, Aleksandra Różyk, Ewa Więsik-Szewczyk

**Affiliations:** 1 Department of Radiology, Military Institute of Medicine, Szaserów 128, 04-141 Warsaw, Poland; azegadlo@wim.mil.pl (A.Z.); mzabicka@wim.mil.pl (M.Ż.); 2 Department of Internal Medicine, Pulmonology, Allergy and Clinical Immunology, Military Institute of Medicine, Szaserów 128, 04-141 Warsaw, Poland; ewiesik-szewczyk@wim.mil.pl

**Keywords:** dual-energy CT, Gemstone Spectral Imaging, spectral CT, virtual monochromatic image, spectral Hounsfield unit attenuation curves, iodine concentration map, solid pulmonary nodule

## Abstract

For some time, dual energy computed tomography (DECT) has been an established method used in a vast array of clinical applications, including lung nodule assessment. The aim of this study was to analyze (using monochromatic DECT images) how the X-ray absorption of solitary pulmonary nodules (SPNs) depends on the iodine contrast agent and when X-ray absorption is no longer dependent on the accumulated contrast agent. Sixty-six patients with diagnosed solid lung tumors underwent DECT scans in the late arterial phase (AP) and venous phase (VP) between January 2017 and June 2018. Statistically significant correlations (*p* ≤ 0.001) of the iodine contrast concentration were found in the energy range of 40–90 keV in the AP phase and in the range of 40–80 keV in the VP phase. The strongest correlation was found between the concentrations of the contrast agent and the scanning energy of 40 keV. At the higher scanning energy, no significant correlations were found. We concluded that it is most useful to evaluate lung lesions in DECT virtual monochromatic images (VMIs) in the energy range of 40–80 keV. We recommend assessing SPNs in only one phase of contrast enhancement to reduce the absorbed radiation dose.

## 1. Introduction

X-rays (discovered in 1895 by W.C. Roentgen) [1] are produced when fast-moving electrons expelled by the cathode in the X-ray tube collide with the anode.

As a result, two different radiation types are generated: braking radiation (Bremsstrahlung radiation) depending on the lamp voltage (keV) and characteristic radiation, where the energy of the photon is characteristic of the anode material.

An essential feature of both types of radiation is penetration, i.e., the ability to pass through matter. This feature depends on the energy of the emitted photons and the atomic structure of the material. In medical applications, the attenuation of radiation passing through the tissue occurs due to two processes: (1) Compton scattering and (2) the photoelectric effect.

**Figure 1:**
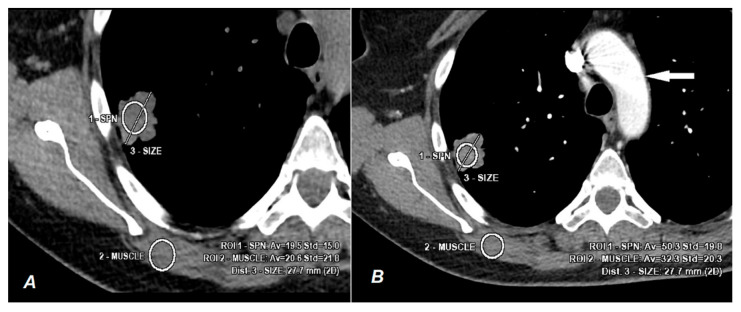
The CT scan shows the measurement of radiation absorption by the pathological tissue of a 27.7-mm lung tumor (ROI 1-SPN) and physiological muscle tissue (ROI 2-muscle) in the same patient based on the Compton effect (**A**) and the photoelectric effect (**B**) caused by the passage of a contrast agent (the contrast medium in the aorta is marked with a white arrow). Image A shows the native phase of CT, and the obtained results of radiation absorption by the tumor (19.5 HU) and muscle (20.6 HU) tissue make it impossible to show differences due to similar penetration of both tissues. Image B, based on the interaction of the X photon with the iodine atom at the energy level of 33.2 kV, shows a clearly greater absorption of X-ray energy by the tumor (50.3 HU) compared to the muscle tissue (32.3 HU). The white arrow indicates contrast medium in the aorta.

A signal dependent on the photoelectric effect is desirable in radiological diagnostics. Human tissues are composed of atoms with a relatively low atomic number (Z), and the energy (K-edge) needed to cause the photoelectric effect in their atoms is in clinical practice too low to achieve proper contrast resolution of individual tissues. The solution is the use of contrast agents, which have the ability to induce proper interaction with photons of ionizing radiation (Figure 1B).

Compton scattering decreases contrast resolution and increases noise in the image; for this reason, it is often impossible to differentiate lesions, as histologically different tissues in the native CT examination may have a similar ability to absorb ionizing radiation (Figure 1A).

Examples of atoms and K-edge energy values as well as the biological effect of photon interactions with their matter are presented in Table 1 [2]. The probability of such interaction is clearly dependent on both the atomic composition of matter (Z) and the energy of the X-ray photon (E). These dependencies are determined by the formula:

Phe = (Z/E)^3^ (1)

In the 1920s, scientists such as Sicard, Forestier, and Moniz searched for methods to increase the diagnostic value of radiological images. The particular aim of their research was to find the optimal substance that is opaque to X-rays and that can be introduced to brain vessels. These agents were based on lithium bromide, strontium bromide, and sodium iodide. Moniz’s achievements resulted in the introduction of the iodine compound to radiological diagnostics, for which he was awarded the Nobel Prize in 1949 [3]. To date, iodine (I) remains a key component of modern contrast agents in CT examinations due to its safety and high efficiency in improving tissue contrast.

Considering how the photoelectric effect occurs in the human body, a number of studies are currently being conducted on alternative, highly effective contrast agents in CT based on gadolinium (Gd, Z = 64, K-edge 50.2 keV [4,5] or tungsten (Z = 74, K-edge 69.5 keV) [6]. The aim of these studies is to search for the optimal balance between improving the resolution of CT imaging and safety. In fact, it is about finding the optimal use of the photoelectric effect in an as large as possible range of CT scanning energy.

We believe that thanks to the DECT modality with Gemstone Spectral Imaging (GSI) analysis, it is possible to analyze this issue and to assess when the use of a contrast agent is actually effective. DECT technology has an established position and is used both to study the composition of various substances [7], for example, to examine urinary calculi [8,9], in noninvasive differentiation of minerals in periarticular soft tissues [10,11] and for automatic bone removal in brain angiography [12]. The GSI system is a single-source DECT system that uses a rapid voltage oscillation with a time of 0.5 ms to generate two different energy levels of 80 and 140 keV [13].

**Figure 2:**
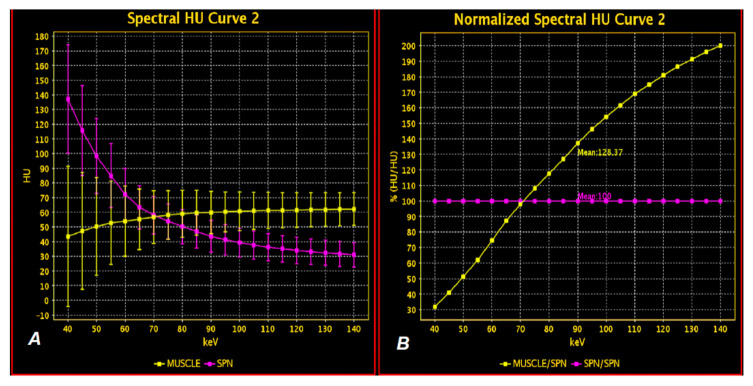
DECT examination. Analysis of X-ray absorption by muscle tissue (yellow) and lung tumor (purple) on graphs showing ionizing radiation absorption curves depending on the photon energy in the range of 40–140 keV with an interval of 5 keV based on actual values (**A**) and normalized values (**B**). Note the intersection of the curves in both graphs A and B at the scan energy levels of approximately 70 keV, which indicates that both muscle and pathological tissue absorb radiation after contrast enhancement in a very similar way at the energy level used in conventional CT. VMI maps enable the assessment of radiation absorption at other levels of radiation energy used to scan both tissues and demonstrate differences based on quantitative data.

The unique structure of the detector based on the properties of the garnet crystal enables separation of the photon energy of both beams (80/140 keV). As a result, it is possible to create X-ray matter absorption curves in HU in the energy range of 40–140 keV [14]. The obtained monochrome images (VMIs) are a computer simulation of what the real image would look like if the study were obtained by monochromatic radiation with a beam of photons with an energy in the range of 40–140 keV, while VMI images with an energy of 65–70 keV are considered comparable to those obtained by a standard CT [15,16,17]. Higher imaging energies reduce artifacts from metallic structures (prostheses, implants) and are mainly used in dentistry and orthopedics [18]. The greatest advantage of VMI maps is the ability to differentiate tissues at photon energy levels other than 70 keV, which corresponds to the image obtained by scanning in a conventional polychromatic beam CT. Examples of measurements of absorption at different radiation energy levels are shown in (Figure 2A,B).

**Figure 3:**
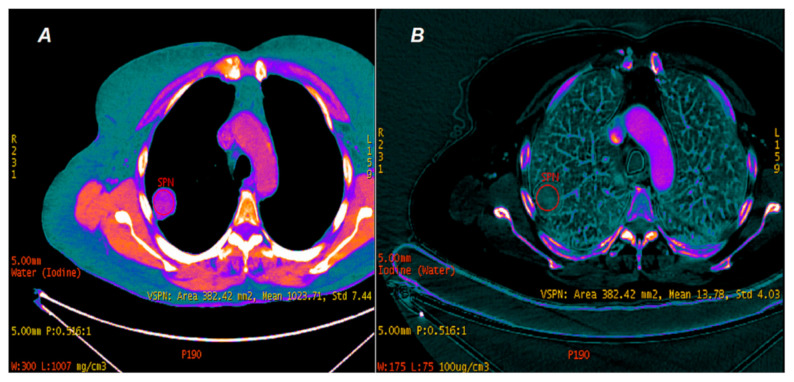
DECT. Lung tumor analysis on maps depending on water (**A**) and iodine (**B**) concentrations. The concentration of water in the lung tumor (SPN) was 1023.71 ± 7.44 mg/cm^3^, and the concentration of accumulated iodine from the contrast agent in the vessels of the same lung tumor was 13.78 ± 4.03 × 100 µg/cm^3^. Both analyses are unique capabilities of the DECT modality.

Another unique feature of DECT is creating concentration maps dependent on preprogrammed substrates such as water on water concentration (WC) images and iodine on iodine concentration (IC) maps (Figure 3A,B).

Postprocessing by subtracting the signal, e.g., from iodine atoms, allows the creation of virtual images without contrast enhancement, thus simulating a classic CT examination without a contrast agent, which is associated with a significant reduction in the dose of absorbed radiation [19,20].

We used iodine concentration-dependent (IC) maps to assess when the absorption of X-rays by soft tissues is no longer dependent on the accumulated iodine contrast and thus how to interpret DECT images. Each focal lung lesion was examined twice (in the arterial and venous phases) after administration of an iodinated contrast agent according to the same scanning protocol. The conclusions can be used to optimize the scanning technique and reduce the patient’s exposure to radiation.

## 2. Materials and Methods

### 2.1. Patients

Between January 2017 and June 2018, DECT scans were performed in 98 patients who were diagnosed with a solid lung tumor and who had not yet undergone treatment. In this study, we analyzed 66 of those patients, all of whom had a histopathologically confirmed diagnosis of a lung nodule. The characteristics of the studied group are presented in Table 2.

The inclusion and exclusion criteria are shown in Table 3. The study was conducted in accordance with the Declaration of Helsinki and approved by the Bioethics Committee of the Military Institute of Medicine in Warsaw, Poland (protocol code 4/WIM/2017, date of approval 17 January 2017). All patients provided written informed consent.

### 2.2. CT Scanning Parameters

The patients underwent examinations on a dual energy DECT scanner Discovery CT 750 HD (GE Healthcare, Waukesha, WI, USA) in the late arterial phase (AP) and venous phase (VP) with delay times after contrast media injections of 35 and 90 s, respectively. The following DECT parameters were set: tube voltage range, 80–140 kVp; tube rotation time, 0.5 s; tube current 630 mA; helical pitch, 1.375:1; large field of view (SFOV), 50.0 cm; collimation, 55 mm; and slice thickness, 0.625 mm. A nonionic contrast medium (400 mg/mL, 1 mL/kg, Iomeron, Bracco Imaging Deutschland GmbH based in Konstanz, Germany) was administered to each patient at a rate of 4.0 mL/s followed by 20 mL of saline flushing with a rate of 3 mL/s using a power injector.

### 2.3. Data Analyses

**Figure 4:**
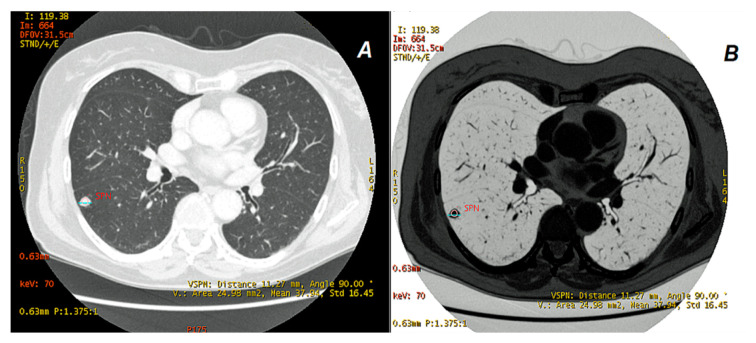
Arterial phase in DECT. Solitary pulmonary nodule (SPN) in the lower lobe of the right lung is marked in red. Measurements of lesion size (11.27 mm) are made on a 70 keV linear gray map (**A**) and additionally on an inverted gray map (**B**), which separates the lesion more clearly from the surrounding lung parenchyma. X-ray absorption (+37.94 ± 16.45 HU) makes the nodule clearly visible on the pulmonary window.

The examinations were processed at Workstation AW (GSI Viewer, GE Healthcare, Waukesha, WI, USA). Two radiologists (M.Ż. and A.Z. with 25 and 15 years of experience in lung imaging, respectively) made measurements independently, and the differences were sorted out by consensus. ROIs were used to measure the average iodine concentration in lung tumors on IC maps (100 µg/cm^3^), WC (water concentration) (mg/cm^3^), and mean X-ray absorption (HU) on virtual monochrome maps (VMIs) in the energy range of 40–140 keV in the late arterial (AP) and venous (VP) phases, respectively. ROIs were measured on 70 keV maps on relatively homogeneous tumor areas while avoiding calcifications. The tumor size was measured along its long axis, in accordance with recommendation [21], while using a pulmonary window (window width (W): 1500 UH, window level (L): 600 HU), Colormaps Linear gray and Inverse gray, as shown in Figure 4.

**Figure 5:**
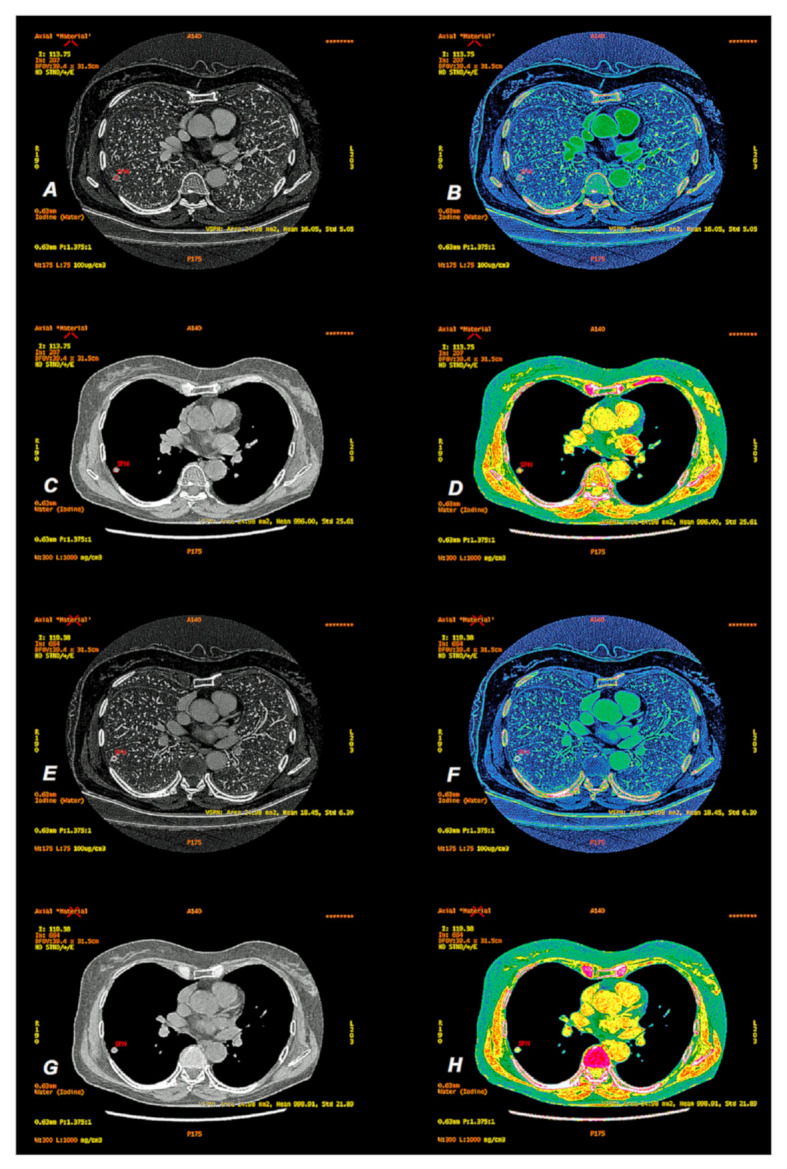
Peripherally located tumor of the right lung (SPN). (**A**,**E**) show images depending on the concentration of iodine (IC) in the arterial (AP) and venous (VP) phases of the contrast enhancement in the linear gray option and their modification on the Colormap French map to improve the contrast of details (**B**,**F**). The iodine concentration in the lesion is 16.05 ± 5.05 (×100 µg/cm^3^) in AP and 18.45 ± 6.30 (×100 µg/cm^3^) in VP. Water concentration-dependent (WC) images are the result of contrast medium subtraction, and as virtual native images (VNC), they can replace native scanning in conventional CT. (**C**,**G**) show examples of iodine subtraction in AP and VP on standard Colormap Linear gray and French modification (**D**,**H**). The SPN measurement values were 996 ± 25.61 mg/cm^3^ (AP) and 998.91 ± 21.89 mg/cm^3^ (VP).

Measurements of iodine concentration (IC) in the window (W: 175 HU, L: 75 HU) and water concentration (WC) in the window (W: 300 HU, L; 1000 HU) in SPNs in the AP and VP phases of contrast enhancement were made with overlays of Colormap Linear Gray and French to increase the contrast of the assessed lesion. Examples are shown in Figure 5.

**Figure 6:**
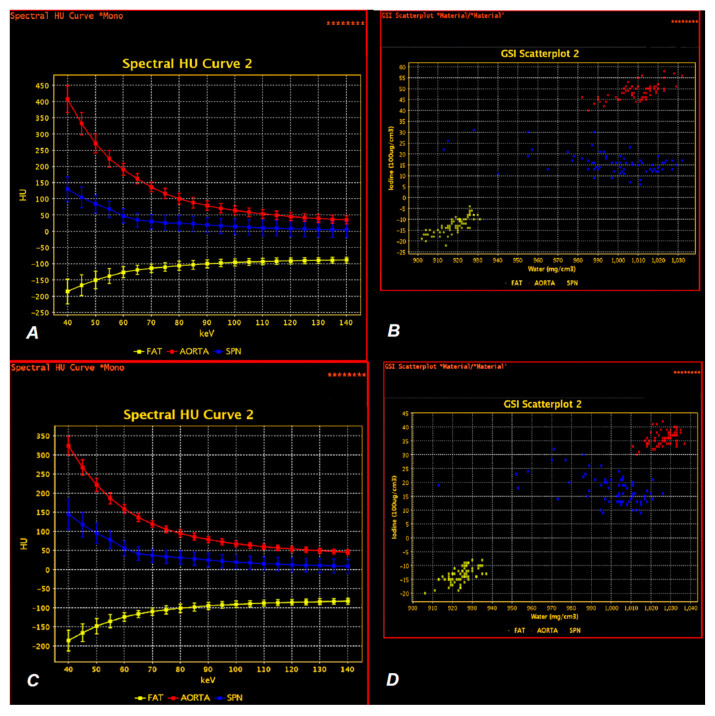
Spectral curves of X-ray absorption by the SPN in the energy range of 40–140 keV in the late arterial phase of contrast enhancement (AP) and venous (VP) phase (**A**,**C**, respectively). The aortic enhancement curve (red) has a similar shape in both phases of contrast enhancement but shows lower measurement values in VP compared to AP, as also shown in scatterplots AP and VP (**B**,**D**, respectively). Both plots (**B**,**D**) show the point distribution of iodine (100 µg/cm^3^) and water (mg/cm^3^) in the ROIs. Photon energy absorption by lung tumor (SPN) measurements is shown in blue. There is a more vertical course of the spectral curve in VP (**C**) in the energy range of 40–70 keV compared to the curve in AP (**A**) and lower scattering of the measurements in VP on the scatterplot (**D**) compared to the results from the AP phase, which were presented in B. The measurements of X-ray energy absorption by the subcutaneous adipose tissue were marked in yellow, and there were no significant differences in the shape of the spectral curves in either phase of contrast enhancement. Red—aortic enhancement, blue—solid pulmonary nodule (SPN), yellow—fat.

The spectral curves of photon energy absorption in the spectral examination were determined based on the MD Analysis/Spectral HU Curve * Mono (keV 70) application in the energy range 40–140 keV, with a 5 keV interval in the AP and VP phases. An example is shown in Figure 6.

The slopes (λ) of the spectral attenuation curves were calculated as the attenuation difference at two energy levels (CT40 keV and CT140 keV) according to the formula in AP and VP, respectively.

λ = (CT40 keV − CT140 keV)/100(2)

### 2.4. Statistical Analysis

Statistical 13.3 software for Windows (TIBCO Software Inc., Palo Alto, CA, USA) was used for the statistical analyses. We used the Lilliefors test to analyze the normality of the distribution of the variables. Variables with a normal distribution are presented as the means and standard deviations. Student’s *t*-test was used to measure normally distributed variable differences, and Pearson’s product-moment correlation coefficient (R) was used to study the relationships between the iodine concentration and material absorption in the AP and VP. Variables with a non-normal distribution (“Size”, “WC AP” and “WC VP”) are presented as medians and 95% confidence intervals. The Wilcoxon test was used to measure the non-normal distribution of the variables. Differences were regarded as statistically significant at *p* ≤ 0.001.

## 3. Results

### 3.1. Radiation Dose

We used a fixed scan protocol. Therefore, for each DECT scan, the volumetric CT dose index (CTDIvol) was a fixed value of 12.72 mGy in the AP I VP phase. The radiation dose was higher than the standard chest CT scan protocol that is routinely used in our institution (average conventional CT scan values are CTDIvol = 11. 25 mGy), complying with the approved protocol.

#### 3.1.1. Photon Interaction with Iodine Contrast Agent Accumulated in Lung Tumors

The mean iodine concentration (100 µg/cm^3^) and the degree of X-ray absorption (HU) were evaluated at 21 measurement levels in the range 40–140 keV, with an interval of 5 keV. The degree of correlation between the concentration of accumulated iodine and the absorption of photon energy in lung tumors was presented in numerical form, determining the *R* correlation coefficient assuming the statistical significance of the results at the level of *p* ≤ 0.001. Statistically significant results of these correlations were found in the energy range of 40–90 keV (0.83 > *R* > 0.45) in the AP phase and in the range of 40–80 keV (0.89 > *R* > 0.42) in the VP.

**Figure 7:**
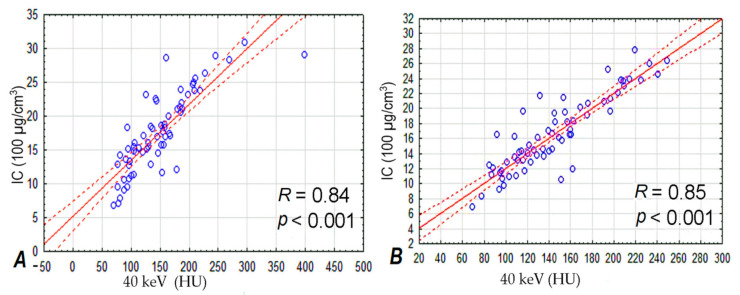
At a scanning energy of 40 keV, the highest level of correlation was found between the concentration of contrast agent in lung tumors (vertical axis of both graphs) and their ability to absorb radiation (horizontal axis) at 35 (**A**) and 90 s (**B**) after intravenous administration of contrast agent.

The strongest correlation was found between the concentrations of the contrast agent and the scanning energy of 40 keV (Figure 7).

**Figure 8:**
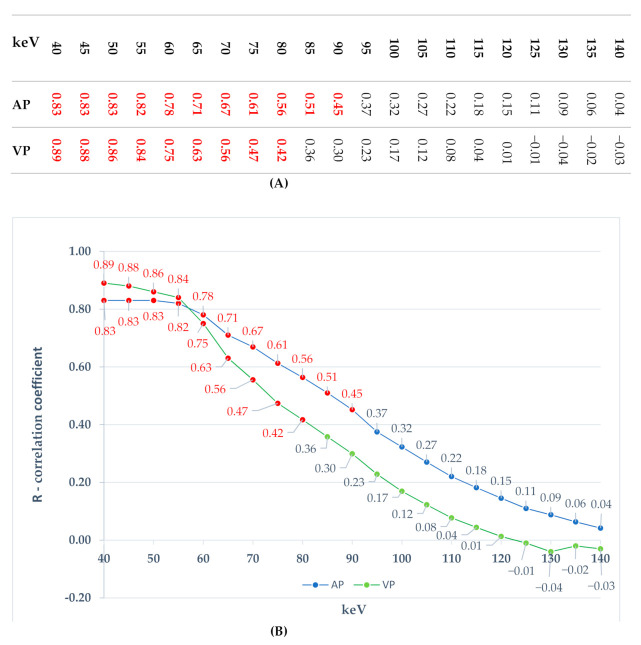
The correlation between the absorption of X-rays and the concentration of iodine in lung tumors. The test was performed using the DECT spectral technique in the emission energy range of 40–140 keV, with an interval of 5 keV (21 measurement levels). Measurements of iodine concentration were made on images dependent on the concentration of iodine atoms (IC) 35 s (AP) and 90 s (VP) after administration of the intravenous contrast agent. The results are presented in the form of a table (**A**) and a chart (**B**) based on the R-Pearson correlation coefficient. In Figure 8B, statistically significant measurements (*p* ≤ 0.001) are marked in red. The upper curve in the graph shows the results of the R correlation coefficient for the AP phase (blue) and the lower curve for the VP venous phase (green). Their shapes are similar to each other and show a clear downward trend in the degree of correlation and the level of statistical significance of measurements with increasing scanning energy.

In the higher scanning energy ranges of 95–140 keV in AP and 90–140 keV in VP, we also found correlation between their energy and iodine concentration in SPNs; however, this correlation was weaker than what was observed in lower scanning energies. The results of correlation are presented in Figure 8 and in the measurement chart complementing it.

#### 3.1.2. Comparison of Iodine Concentration in IC Maps, Water Concentration (WC) and Radiation Absorption in Lung Tumors in AP and VP of DECT Examination

**Figure 9:**
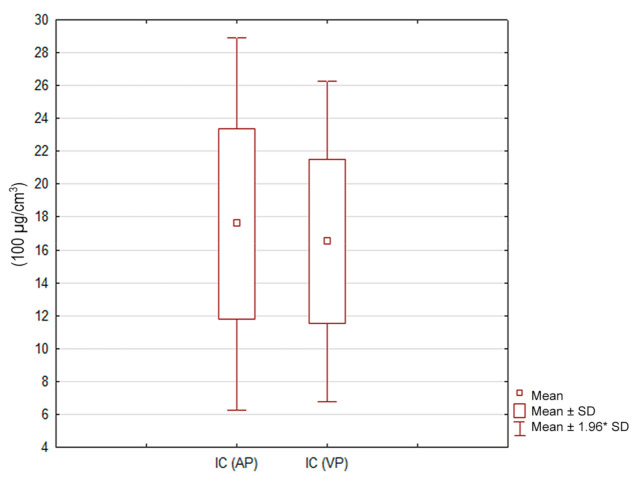
Iodine concentration (100 µg/cm^3^) in lung tumors in the late arterial (AP) and venous (VP) phases. Box-and-whisker plots display the distribution of the results, with no statistically significant differences between the two phases (*p* = 0.25). 1.96* SD―1.96 standard deviations of the mean.

In the venous phase (VP) of the scan, statistically insignificant (*p* = 0.25) and lower mean concentrations of iodine were observed in lung tumors compared to the late arterial (AP) phase (16.5 and 17.6 × 100 µg/cm^3^, respectively) (Figure 9).

**Figure 10:**
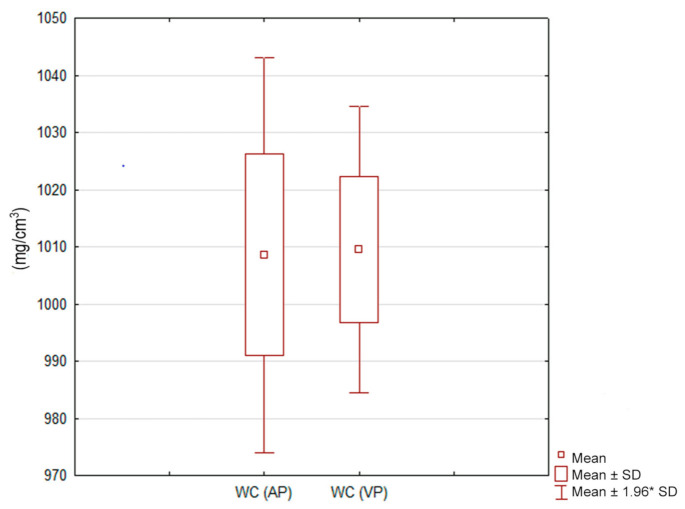
Water concentration (WC) (mg/cm^3^) in lung tumors in the late arterial (AP) and venous (VP) phases. Box-and-whisker plots display the distribution of results with no statistically significant differences between the study phases (*p* = 0.74). 1.96* SD―1.96 standard deviations of the mean.

No significant differences were found between the measurements of water concentration in lung tumors in either scanning phase (*p* = 0.74). WC images are used to measure the concentration of water compounds in ROIs. They are the result of iodine subtraction from voxels in the measurement area and, as images virtually devoid of iodine overlay, they are comparable to native images obtained in conventional CT [22] (Figure 10).

No statistically significant differences were found in the absorption of radiation energy by the examined lung tumors radiated with X-rays in the energy range of 40–140 keV between the arterial and venous phases of contrast enhancement. The results are summarized in Table 4.

**Figure 11:**
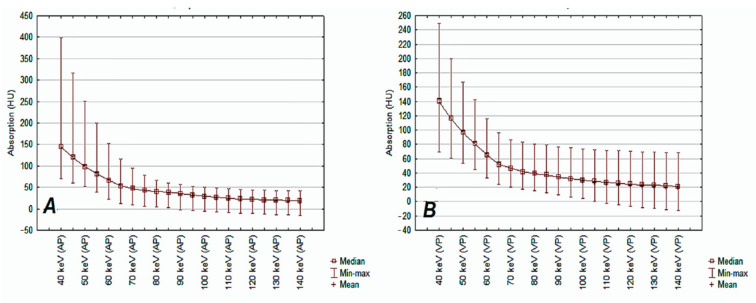
X-ray absorption distributions by lung tumors (HU) are presented as the mean values of tumor tissue enhancement after administration of iodinated contrast agent (vertical axis) at 35 s (**A**) and 90 s (**B**) of DECT scan. The DECT scan energy ranges were 40–140 keV with an interval of 5 keV (horizontal axis of both graphs).

The decrease in the photon energy absorption capacity with increasing X-ray energies in AP and VP was presented in the form of curves (Figure 11) The median of the λ slope curve in AP was 1.23 and was 0.06 higher than the median λ in VP, which indicates a higher amplitude of measurements in AP in the absence of statistically significant differences (*p* = 0.10).

The average age of the respondents was 66 ± 11 years, with the female population being older. Age differences between men (mean 63.8 ± 12.7 y) and women (mean 68.5 ± 8.3 y) were not statistically significant (*p* = 0.11). There was also no statistical significance of the low correlations between the concentration of iodine in lung tumors and age and body weight in the whole patient group (*n* = 66), as well as separately in the group of men (*n* = 43) and a smaller group of women (*n* = 23). Those results are presented in Table 5.

## 4. Discussion

The formation of new vessels to supply nutrients and oxygen promotes tumor growth and is a marker of tumor viability used in assessing the response to therapy [23]. According to Swensen et al., the accumulation of iodine in the tumor vessels resulting in contrast enhancement by more than +20 HU compared to native density allows the assessment of tumor malignancy using the CE-CT method with a sensitivity of 95–100%, a specificity of up to 70–86%, and accuracy at the level of 85–97% [24]. Dynamic CT and MRI, PET, and SPECT examinations, which are the other diagnostic methods that benefit from contrast-enhanced images, allow for the differentiation of lung tumors with a similar percentage of false results, regardless of the method used [25].

DECT, used for the first time for clinical applications in the diagnosis of lung tumors in 2008 [26], allows the differentiation of tumors at the initial stage of their clinical advancement [27]. Based on the theoretical basis of inducing a photoelectric effect in matter, we have demonstrated in practice that the intravenous administration of an iodine contrast agent has an impact on the evaluation of focal lesions in DECT based on the analysis of iodine concentration, provided that the appropriate level of scanning energy is used.

In the DECT study with GSI spectral analysis, the requirement for using maps depending on the atomic composition is that the examined matter or the contrast material in the studied area has spectral properties—that is, it differs from the “background” in attenuation of X-rays at different photon energies. Maps depending on the concentration of iodine administered as a contrast agent in DECT (IC) are widely used in oncology for diagnostics [28,29,30], perfusion assessment [31,32,33], and prognosis [34]. Since the iodine concentration in lung tumors has the same value depending on the amount of contrast in the tumor vessels on the VMI maps, we used the accumulated iodine atoms to assess their influence on the photon energy absorption and to investigate for which scanning energies the images obtained are strictly dependent on injected contrast agent, and for which it is not the case, which helps to optimize the use of DECT in clinical practice.

Monochrome VMI reconstructions result from scanning with low and high photon energy and are the result of computer simulation of what the obtained scan would look like using a monochrome X-ray beam to the selected predicted or recommended energy level [13]. Our measurements on VMI maps were based on DECT scans in the energy range of 40–140 keV, and the results of photon absorption were correlated with the iodine concentrations in the measurement areas. Based on the current literature, VMI images with energies in the range of 60–70 keV are the most frequently recommended as equivalent to conventional CT images obtained from scanning at 120 kVp acquisition [35,36,37,38].

Their usefulness is influenced by both contrast-to-noise ratio (CNR) analysis and the subjective overall quality of imaging reported by radiologists. We performed a usefulness check of DECT images based on the use of the interaction of iodine atoms and photon monoenergy. According to these findings, the acquisition in the photon energy range of 40–90 keV (AP) and 40–80 keV (VP) was highly correlated (*p* < 0.001) with the absorption of radiation by the matter enhanced with iodine contrast.

Simultaneously, in the same tumors, we did not observe a statistical correlation between the higher photon energies used to X-ray the tumor and the absorption, which means that in statistical terms, it is not directly dependent on the concentration of iodine in the tumor vessels. In the case of the lowest DECT scanning energy (40 keV), the high correlation coefficient R with absorption indicates the high clinical utility of VMI in the diagnosis of focal lesions based on angiogenesis. In recent years, a significant number of publications have appeared indicating the use of low-energy reference VMI images in oncological diagnostics, which is consistent with our results.

Among others, Liu et al. indicated the best contrast-noise-ratio (CNR) values, the best subjective gastric wall assessment, and the best diagnostic accuracy in identifying parietal infiltration in 40 keV imaging in comparison to traditional CE-CT images with 120 kVp acquisition [39]. Forghani et al. reported that VMI imaging at the level of 40 keV improves the visibility of the tumor objectively and subjectively, both as assessed by specialists in head and neck radiology and by general radiologists [40]. The authors of a 2020 study on hypodense liver metastases reported that noise-optimized VMI significantly improved the contrast and imaging of liver metastases in patients with fatty liver compared to standard reconstruction, emphasizing the role of 40 keV imaging [41].

An additional advantage of using DECT imaging is the sustained effect of X-ray energy absorption during observation up to 90 s after the administration of the contrast agent. The benefit of this is that the scan is only performed in one predetermined contrast phase. This correlation was observed over the entire energy spectrum used to scan lung nodules (40–140 keV). This effect was also observed by other authors who investigated the usefulness of VMIs in the assessment of urothelial cancer [42].

They demonstrated the usefulness of VMI excretory phase 40 keV images compared to the venous phase of CE-CT scanning, demonstrating higher vascular and parenchymal CNR and similar diagnostic accuracy in the assessment as the venous phase of CE-CT. The authors also reported that 40 keV VMI imaging was effective in preventing the decline in imaging quality of contrast-enhanced structures in the secretory phase of CT. It seems that on VMI maps, the contrast enhancement of tissues is visible for the longest time, even after 240 s. As the correlation between photons and iodine concentration occurs only in the energy range of max 80–90 keV and lower, we suggest that the changes requiring differentiation based on DECT spectral curves should be differentiated in this energy range only, which may help to optimize their accuracy. The imaging of lesions on VMI maps with energy higher than 90 keV is not useful in the assessment of angiogenesis. However, high energies that penetrate through soft tissue can be used to level artifacts from implants [43] and prostheses [44] and to assess bone structures at the tissue border in cases of suspected tumor infiltration that are unresolved in conventional images [18].

Conducting measurements of iodine concentration in two phases, 35 and 90 s after contrast administration, our study showed no significant differences in these measurements in patients with a solid lung nodule regardless of its benign or malignant nature. However, this thesis should be approached carefully, as we believe that the method of selecting the study group and using the DECT protocol is crucial. The results obtained in the particular oncologic protocol are ambiguous. In the evaluation of patients with non-small cell lung cancer (NSCLC), some researchers did not find a significant difference between the arterial and venous phases in the evaluation of NIC and λ_HU_ in patients with low- and high-grade NSCLC, but they also found that ROC analysis (area under the ROC curve (AUC)) indicated that λ_HU_ in the VP provided the best diagnostic performance in distinguishing high-grade cancers from low-grade cancers [45]. On the other hand, Zhang et al., in their work published in 2020, carried out two homogeneous study groups of patients with NSCLC, lung adenocarcinoma, and squamous cell carcinoma, and they showed the potential of DECT in differentiating the type of NSCLC during early VP only but not in AP [46]. However, Li et al. found significant differences in iodine concentration at 40 kV in patients with NSCLC depending on the expression of vascular endothelial growth factor (VEGF), one of the stimulators of tumor angiogenesis, and a positive correlation with the level of VEGF expression in both the AP and VP [47]. In our opinion, in addition to the size of the examined tumor and the selected delay for AP and VP, the proper selection of energy levels used to evaluate the iodine concentration could be the main reason for the differences in the obtained results.

### Limitations

Not unlike other studies regarding this topic, our study has some limitations. The main limitation would be the relatively small study group.

Another significant issue would be the possibility of human error when manually copying ROIs between AP and VP images. We tried to minimize that by being as thorough as possible.

## 5. Conclusions

In spectral scanning of lung tumors, the absorption of photon energy by the examined tissue based on the iodine contrast agent occurs in the low emission energy range and is the highest at 40 keV. The differential evaluation of the contrast enhancement of lung lesions based on the spectral curves could be performed on VMIs 40–90 keV in the arterial phase or 40 keV–80 keV in the venous phase of contrast enhancement. In clinical practice, to assess the degree of contrast enhancement of lung nodules, it is recommended to use VMI images in the emission energy range of 40–80 keV, regardless of the selected DECT scanning phase. Concerning the safety of the patients, assessment of the characteristics of lung tumors in only one phase of iodine contrast enhancement provides a significant amount of diagnostic information, allows a reduction in the dose of absorbed radiation, and contributes to the optimization of work by reducing the amount of data stored on archiving devices. Absorption of X-rays by human matter at a high photon energy level > 90 keV does not depend on its enhancement with intravenous iodine contrast agent.

## Figures and Tables

**Table 1:** The atomic number (Z) of the most common atoms of the human body and the corresponding energy necessary to remove an electron from the K shell. The asterisk (*) refers to atoms that are components in selected contrast agents used in radiological diagnostics, enabling the formation of X-ray absorption based on the photoelectric effect.

Atom	Z Number	K-Edge Energy (keV)	Absorption
Hydrogen (H)	1	1.36000 × 10^−2^	Compton scattering
Carbon (Ca)	6	2.83800 × 10^−1^	Compton scattering
Nitrogen (N)	7	4.01600 × 10^−^^1^	Compton scattering
Oxygen (O)	8	5.32000 × 10^−^^1^	Compton scattering
Fluorine (F)	9	6.85400 × 10^−^^1^	Compton scattering
Sodium (Na)	11	1.07210 × 10^0^	Compton scattering
Magnesium (Mg)	12	1.30500 × 10^0^	Compton scattering
Potassium (K)	19	3.60740 × 10^0^	Compton scattering
Calcium (Ca)	20	4.03810 × 10^0^	Compton scattering
Iron (Fe)	26	7.11200 × 10^0^	Compton scattering
Iodine (I) *	53	3.31694 × 10^1^	Photoelectric effect
Barium (Ba) *	57	3.74406 × 10^1^	Photoelectric effect
Gadolinium (Gd) *	64	5.02391 × 10^1^	Photoelectric effect

**Table 2:** Characteristics of the studied group and size of tumors assessed by DECT in millimeters. SD—standard deviation.

Patients (*n* = 66)		
Age (y), mean ± SD	66 ± 11	
Sex (male/female)	43/23	
Size (mm) ± SD	21.2 ± 7	
Histopathology (*n*)	Adenocarcinoma	22
	Squamous cell carcinoma	18
	Inflammatory infiltrations	11
	Sarcoidosis	5
	Large cell neuroendocrine carcinoma	3
	Fibroma	2
	Squamous cell (95%) and neuroendocrine ca.	1
	Small cell carcinoma	1
	Hamartoma	1
	Hematoma	1
	Tuberculoma	1

**Table 3:** Inclusion and exclusion criteria.

Inclusion Criteria	Exclusion Criteria
98 patients with a solid lung tumor Written consent for DECT examination with arterial and venous phase (AP and VP)	Exclusion criteria for CT examination (*n* = 14)
	Family history of lung cancer (*n* = 5) Kidney failure (*n* = 5) Contrast media hypersensitivity (*n* = 3) Lack of patient’s consent (*n* = 1) Pregnancy (*n* = 0)
84 patients	Exclusion criteria for analysis (*n* = 18)
	Lesion of a long-axis diameter larger than 30 mm (*n* = 8) “Ground-glass” lesion (*n* = 6) Lack of histopathological confirmation of diagnosis (*n* = 4)
66 patients	

**Table 4:** There were no statistically significant differences between the mean values of IC, WC, and energy absorption by lung tumors measured on VMI in the AP and VP phases. (*) Variables with non-normal distribution.

Parameter	Time 35 s. (AP) Mean ± SD * Median, 95% CI		Time 90 s. (VP) Mean ± SD * Median, 95% CI		*p*-Value
IC (100 µg/cm^3^)	17.6	5.8	16.5	4.98	0.25
WC (mg/cm^3^)	1010.6 *	15–21.3 *	1012 *	10.9–15.4 *	0.74
40 keV	149.1	58.3	144.5	44.3	0.17
45 keV	122.3	46.6	119.0	35.4	0.06
50 keV	100.7	37.4	98.6	28.5	0.10
55 keV	84.1	30.5	82.8	23.4	0.39
60 keV	67.6	25.1	66.8	19.5	0.53
65 keV	55.8	21.4	55.4	17.2	0.71
70 keV	49.3	18.5	49.3	15.5	0.99
75 keV	44.0	16.5	44.3	14.3	0.91
80 keV	40.1	15.1	40.8	13.5	0.78
85 keV	36.6	14.5	37.5	13.0	0.72
90 keV	32.9	13.9	34.2	12.8	0.58
95 keV	29.7	13.5	31.3	12.7	0.48
100 keV	27.5	13.4	29.0	12.7	0.50
105 keV	25.5	13.3	27.1	12.8	0.46
110 keV	23.6	13.3	25.4	12.9	0.44
115 keV	22.2	13.3	24.0	12.9	0.42
120 keV	20.9	13.3	22.8	13.0	0.41
125 keV	19.9	13.2	21.8	13.1	0.42
130 keV	18.8	13.4	21.0	13.4	0.35
135 keV	17.9	13.3	19.9	13.3	0.26
140 keV	17.4	13.3	19.3	13.3	0.39

**Table 5:** Pearson correlation of the IC SPN variable depending on the age and body mass parameters for all subjects and by gender. AP—arterial phase, VP—venous phase, IC SPN—iodine concentration in solitary pulmonary nodule, *R*—correlation coefficient, *R*^2^—coefficient of determination (*R* squared), *p*-value—statistical significance.

Correlation	*R* =		*R*^2^ =		*p*-Value
IC SPN (AP) (100 µg/cm^3^)/ Age (Y)	All	0.29	All	0.08	0.018
	Man	0.23	Man	0.05	0.133
	Woman	0.33	Woman	0.10	0.127
IC SPN (AP) (100 µg/cm^3^)/ Body mass (kg)	All	0.12	All	0.02	0.326
	Man	0.03	Man	0.00	0.848
	Woman	0.12	Woman	0.01	0.577
IC SPN (VP) (100 µg/cm^3^)/ Age (Y)	All	0.32	All	0.10	0.007
	Man	0.27	Man	0.07	0.074
	Woman	0.33	Woman	0.11	0.118
IC SPN (VP) (100 µg/cm^3^)/ Body mass (kg)	All	0.15	All	0.02	0.215
	Man	0.02	Man	0.00	0.914
	Woman	0.09	Woman	0.00	0.694

## Data Availability

Patient data that we based our results on is confidential and not available publicly.
